# Boosting brain tumor segmentation: A novel 3D pooling approach with U-net 3D

**DOI:** 10.1371/journal.pone.0336514

**Published:** 2026-02-02

**Authors:** Mohamed Gasmi, Mohammed Elbachir Yahyaoui, Makhlouf Derdour, Hakim Bendjenna, Yazeed Alkhrijah, Wojdan BinSaeedan, Waad Alhoshan

**Affiliations:** 1 Laboratory of Mathematics, Informatics and Systems, Echahid Cheikh Larbi Tebessi University, Tebessa, Algeria; 2 Department of Computer Science, Larbi ben Mhidi University, Oum El Bouaghi, Algeria; 3 Imam Mohammad Ibn Saud Islamic University (IMSIU), Riyadh, Saudi Arabia; Dayananda Sagar University, INDIA

## Abstract

Brain tumor segmentation is a crucial task in medical imaging that has a significant impact on diagnosis and treatment planning. This study introduces a novel 3D pooling layer within the U-Net 3D architecture to enhance segmentation accuracy from multimodal MRI. The method addresses the limitations of conventional pooling techniques by considering the interdependencies between MRI pixels, thereby improving the model’s ability to capture complex tumor structures. To increase robustness to intensity variation, two complementary normalization pipelines were trained independently with identical networks, and predictions from selected epochs were fused by simple probability averaging to form the final ensemble. Evaluation was conducted on BraTS2020 using five-fold cross-validation. On the validation set, the ensemble achieved Dice (ET/TC/WT)=0.8299/0.8882/0.8986 and HD95=4.40/4.95/11.14, reflecting consistent gains over max-pooling variants and comparing favorably with recent methods while using a lightweight fusion mechanism. These results confirm the effectiveness of the proposed 3D pooling approach and pave the way for more robust algorithms in automated brain tumor segmentation.

## Introduction

Medical image processing is a specialized field of study that applies various techniques and algorithms to images obtained through diverse imaging modalities such as X-ray, computed tomography (CT), magnetic resonance imaging (MRI), ultrasound, and positron emission tomography (PET), to improve their quality, analyze content, and extract valuable information [1]. Different methods are employed to enhance image quality, segment and classify structures and tissues, detect and diagnose abnormalities and diseases, and monitor disease progression over time [2]. This field finds numerous applications in clinical practice, research, and education, playing a vital role in modern healthcare.

**Fig 1:**
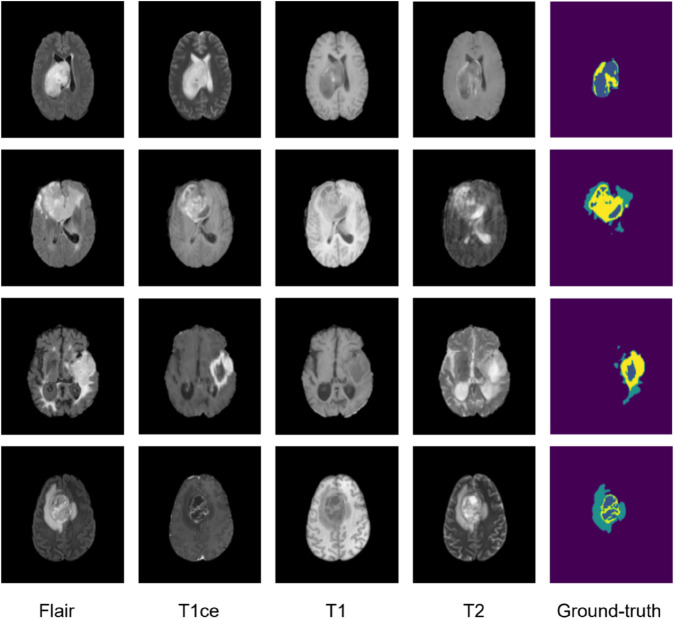
Examples of the BraTS 2020 dataset on brain tumors images. Yellow: enhancing tumor (ET), Blue: non-enhancing tumor/necrotic tumor (NET/NCR), Green: peritumoral edema (ED) [4].

Brain tumors are abnormal growths of cells in the brain, which are classified as either benign (noncancerous) or malignant (cancerous) [3]. They can originate from several types of brain cell, including glia, neurons and meninges. Symptoms depend on the location and size of the tumor, such as headaches, nausea, blurred vision or hearing, memory loss and personality changes. According to the latest statistics from the American Cancer Association, brain tumors are estimated to account for around 25,400 new cases of cancer of the brain and other nervous systems in the United States, representing around 1.3% of all newly diagnosed cancer cases [3]. Medical imaging is the primary diagnostic tool, whether by magnetic resonance imaging (MRI) or computed tomography (CT), with pathological confirmation by biopsy. Positron emission tomography (PET) is also useful for diagnosing brain tumors. MRI, using multiple imaging modalities, is the main diagnostic method. Multi-modality MRI scans provide complementary information, enabling efficient tumor analysis [4] (see Fig 1). Robust delineation of glioma sub-regions-enhancing tumor (ET), tumor core (TC), and whole tumor (WT)-directly informs surgical planning and radiotherapy targeting, making reliable automatic segmentation clinically consequential.

The aim of brain tumor segmentation is to divide the tumor region into several subregions, such as contrast tumor (CT), peri-tumor edema (PE) and necrotic or non-enhanced tumor nucleus (NECT), to facilitate treatment planning and monitoring of disease progression. Deep learning is considered the most effective approach for segmentation in general and brain tumor segmentation in particular, where a deep neural network is trained to automatically segment brain tumors from MRI scans.

Two persistent barriers limit reliability in practice: (i) standard max-pooling discards local relationships during down-sampling, weakening boundary fidelity; and (ii) inter-site intensity heterogeneity hampers cross-center generalization. To address these gaps, this study integrates a Related 3D Pooling operator into a 3D U-Net backbone to preserve inter-voxel context and employs two complementary normalization pipelines-min-max (1st-99th percentile) and z-score on non-zero voxels-trained with identical architectures; predictions from selected checkpoints are fused by simple probability averaging to form a lightweight ensemble.Evaluation is conducted on BraTS-2020, a heterogeneous multi-institution clinical MRI benchmark comprising 369 multi-contrast scans from 19 institutions, using five-fold cross-validation; on the validation set, the ensemble achieves Dice (ET/TC/WT) = 0.8299/0.8882/0.8986 and HD95 = 4.40/4.95/11.14, exceeding the individual pipelines.

Deep learning and segmentation techniques have seen a significant improvement in the diagnosis of brain tumors, providing accurate and reliable segmentation results that facilitate therapeutic management and monitoring of disease progression. Accurate segmentation of brain tumor subregions is crucial for understanding tumor progression and its impact on nearby structures, which helps guide treatment decisions like surgery, radiotherapy, or chemotherapy [5]. These techniques are considered the most objective and consistent solutions for improved tumor segmentation compared to traditional methods such as manual segmentation and thresholding. Deep learning segmentation is fast, accurate segmentation and less prone to variability, as it relies on the distinctive extraction of features from medical images. It can also help detect brain tumors early, leading to better outcomes through early treatment and reduced risk of complications. Another point provided by deep learning segmentation techniques is their ability to facilitate continuous monitoring of tumor progression, allowing the necessary interventions to be carried out.

The BraTS dataset is widely used in brain tumor diagnosis, providing numerous MRI images for training and testing, making it a valuable resource for evaluating deep learning models.

This dataset is a valuable resource for developing, evaluating, and comparing innovative computer models and algorithms in brain tumor segmentation and classification.

The remainder of the paper is organized as follows: an overview of the existing literature in the field of brain tumor segmentation and related techniques is provided;. Next, the proposed approach is described in detail, discussing the architecture of the neural network, the 3D clustering technique, and the loss function used for training. In the experiments section, we find the discussion the experimental setup, evaluation metrics and implementation details. Next, the presentation of results, offering quantitative and qualitative evaluations of the method. Results are compared with related studies, and ablation experiments assess the contribution of each component of the approach. The paper finish with a conclusion and future research directions.

## Related work

Several works have significantly contributed to improving the metrics used for evaluating the performance of brain tumor segmentation models with BraTS 2020. This re-search is based on a variety of methods and techniques, including network architectures, loss functions, optimizers, and data augmentation strategies

One notable study by Wang et al. [4] focused on modality-pairing learning, aiming to effectively exploit the complementary information from multimodal MRI data using a 3D U-Net architecture. The authors designed parallel branches to extract features from different modalities with a sequence of relations between layers to capture complex connections and ignorant information between modalities. They also used a loss of coherence to minimize the prediction variance between the two branches. In addition, they adopted a learning rate warm-up strategy to eliminate the problem of learning instability and early over-fitting.

Henry et al. [6] proposed a solution based on self-assembled 3D U-net neural networks with deep supervision. the proposed approach involves on training multiple networks with various initializations by applying an ensemble strategy to obtain robust predictions. They relied on stochastic weight averaging (SWA) to minimize overfitting and, by incorporating deep supervision, effectively addressed the gradient vanishing problem and achieving competitive results. Fidon et al. [7] proposed two independent sets of models from two different trained pipelines, each produced a brain tumor segmentation map. These two patient-based labeling maps were then merged, considering the performance of each set for specific tumor subregions.

The proposed work of Nguyen et al. [8] is based on the use of two sets of models (U-Net and nested BiFPN) along with an additional classification network to improve MRI segmentation of brain tumors. The approach consists of training a segmentation model together with a classification model with the aim of differentiating tumoral and non-tumoral regions, thereby improving segmentation accuracy and better differentiating tumor boundaries.

Syazwany et al. [9] introduced a multi-modality fusion network based on the Bi-directional Feature Pyramid Network (Bi-FPN) for MRI brain tumor segmentation. Their model consists of four independent encoders, a Bi-FPN feature fusion, and a shared decoder. Modality-specific features are captured by the encoders, and combined using Bi-FPN layers to fuse multimodal information by exploiting the descending and ascending paths of the feature pyramid. The results of this fusion are fed at the 2th step into the shared decoder to generate the final segmentation image. The aim of this approach is to capture and combine information from different modalities at multiple scales, and thus improve the accuracy of tumor segmentation.

Ding et al. [10] focus their research on the non-availability of all modalities for each patient in the context of multimodal segmentation of brain tumors. Their proposed RFNet region-aware fusion network efficiently handles missing modalities. Based on the encoder-decoder architecture, the region-dependent fusion module (RFM), assigns importance weights to each modality as well as region-level attention, the model efficiently aggregates multimodal information from various regions. Additionally, RFNet incorporates regularization to ensure accurate segmentation by learning discriminative features for all tumor regions.

The CH-UNet brain tumor segmentation model was proposed by Xu et al. [11], where they integrated a corner attention mechanism (CAM) and high-dimensional perceptual loss (HDPL). The purpose of CAM is the efficient modeling of relationships between the sagittal, coronal and axial axes, capturing interslice information and enriching contextual information. HDPL in turn preserves local coherence and explores perceptual similarity, making predictions and ground truth similar in a high-dimensional space. The architecture of the CH-UNet model is based on U-Net, with modifications including group normalization, residual connections, Leaky ReLU, Dropout and deep supervision.

A framework called AGSE-VNet 3D was proposed by Guan et al. [12] for the automatic segmentation of brain tumor MRI data. Their model integrates anisotropic convolutional layers with a new Categorical_Dice loss function to efficient handling of anisotropic voxel sizes on the one hand, and to improve the generalizability of the model on the other. Also, they integrated two modules - the Squeeze and Excite (SE) and Attention Guide Filter (AG)- into the VNet architecture. The aim of the SE module is to reinforce important information in the channel whereas suppressing irrelevant information, while the AG uses attention gates to guide information to the edges and suppress noise.

The MH-UNet architecture, proposed by Ahmad et al. [13] for medical image segmentation, is based on hierarchical multi-scale structuring and the integration of several advanced techniques. The model implements densely connected blocks and residual intercept blocks for to enhance feature extraction. The former block enabled the integration of global and local contextual information at multiple scales. while deep supervision was used to facilitate gradient flow and improve training. A combination of binary cross-entropy and dice loss functions are used for accurate segmentation.

The approach proposed by Silva et al. [14] is based on multi-stage deep layer aggregation for brain tumor segmentation. Their architecture uses a cascade of three deep layer aggregation (DLA) neural networks and integrates multi-sequence MRI images to progressively refine segmentation results, improving accuracy and boundary delineation. The aim of the deep-layer aggregation technique is the semantic and spatial fusion of features. Top-down sampling is performed using max pooling followed by Gaussian filtering, while bottom-up sampling is performed using transposed convolutions. The model is trained with three auxiliary losses and shows promising results in glioma segmentation, overcoming the challenges of tumor heterogeneity and different imaging modalities. The use of Gaussian filters during clustering helps reduced aliasing artifacts.

Lin et al. [15] introduced the Aggregation-and-Attention Network (AANet) for brain tumor segmentation. Their approach utilized a U-Net backbone and incorporated an enhanced down-sampling module (EDS), multi-scale connection module (MSC), and dual-attention fusion (DAF) to extract and aggregate useful semantic information for improved glioma segmentation. The EDS module compensates for information loss and enhances encoding quality. The MSC module replaces skip connections and extracts context semantic information. The DAF module employs dual-attention heads to capture positional and channel attention features.

To avoid high computational costs, recent study of Rajput et al. [16] proposed the optimized triplane method, a lighter version of the 3D U-Net. A three 2D combined architecture with attention mechanism which led to better segmentation result with fewer parameters. Kundal et al. [17] conducted a study on the BraTS2021 dataset to evaluate and compare the effectiveness of four common CNN-based methods for brain tumor segmentation: CaPTk, 2DVNet, EnsembleUNets, and ResNet50. The results indicated that EnsembleUNets outperformed the other methods in both direct segmentation and radiomic feature comparison, making it the most accurate among the tested methods for brain tumor segmentation.

These selected works represent a range of innovative techniques in brain tumor segmentation, including modality-pairing learning, specialized loss functions, additional classification networks, and novel pooling operations. By positioning our work within the context of these prior studies, we demonstrate its unique contribution and highlight its potential impact in further advancing the field of brain tumor segmentation. A recent works [18–20] implement a federated learning (FL) approach with privacy-preserving techniques (PPTs) directed toward segmenting brain tumor lesions in a distributed and privacy-aware manner. They employed a model of 3D U-Net, which is trained using federated learning on the BraTS 2020 dataset. The model achieved DSCs of 89.85%, 87.55%, and 86.6%, with HD95 values of 22.95 mm, 8.68 mm, and 8.32 mm for WT, TC, and ET, respectively. Authors in [21] fuses multiple 3D U-Net variants using coalition game-derived fuzzy measures (Shapley values) with novel channel/width shuffling to enhance spatial detail. On BraTS-2020, it reports strong results-Dice: WT 0.896, TC 0.851, ET 0.792; HD95: WT 5.96 mm, TC 6.65 mm, ET 20.74 mm-surpassing several recent baselines. In [22] BTSegDiff leverages multimodal MRI and a diffusion probability model dynamically guided by extracted features to generate precise brain tumor segmentation masks. It incorporates a Fourier domain feature fusion module to reduce noise impact and a stepwise uncertainty sampling module for unique and accurate results. Validated on BraTS2020 and BraTS2021 benchmarks, the method outperforms many existing segmentation approaches, showing improved accuracy and reproducibility. The study [23] presents a deep ensemble learning framework based on multimodal MRI and a 3D U-Net backbone, designed for simultaneous glioma segmentation and risk grade prediction. The framework incorporates dual-domain attention and asymmetric convolution modules to enhance feature extraction, along with a multi-task loss function to optimize both tasks concurrently. Evaluated on the BraTS2020 dataset, the method achieved superior segmentation accuracy and precise grading performance compared to existing approaches, providing a robust multitask solution for glioma analysis.

In conclusion, although each study presents strengths and contributions to the field of brain tumor segmentation, further research is needed to overcome limitations and further improve the performance of automated segmentation models. By combining the advantages and strengths of different approaches and exploring new techniques, researchers can continue to improve the accuracy and efficiency of brain tumor segmentation methods.

## Materials and methods

Two separate training pipelines were implemented, each using a shared neural network architecture based on the 3D U-Net model, which is a commonly used method for medical image segmentation. The use of separate training pipelines was intended to foster diversity in network predictions, exploring potential variations in performance. This section provides detailed insights into each pipeline, denoted as pipeline A and pipeline B, and underscore the key distinctions between them.

**Fig 2:**
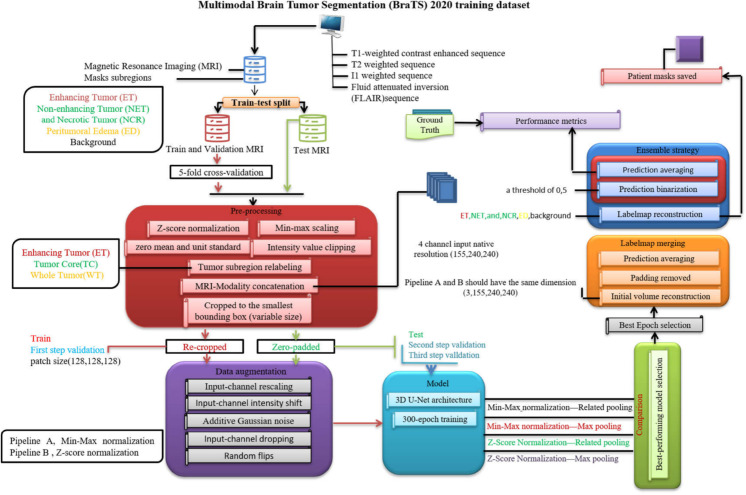
Overall block diagram of the proposed strategy used for enhancing brain tumor segmentation accuracy.

In addition, the study examined how different pooling layers influence the segmentation results, specifying the types of layers used. In conjunction with the conventional max pooling layer, a novel pooling layer termed (related 3D pooling) was introduced. By integrating both max pooling and related pooling, the aim was to assess the impact of pooling strategies on model performance. Subsequent sections will delve into the specifics of each pipeline and elucidate the divergent effects of the pooling layers. Fig 2 illustrates the flowchart of the proposed methodology.

### A. DataSet

The BraTS2020 dataset, a widely recognized benchmark in brain tumor research, comprises a comprehensive collection of 369 multi-contrast MRI scans [24–27]. These scans encompass two major categories: 293 scans from glioblastoma (GBM/HGG) representing high-grade gliomas and 76 scans from lower-grade glioma (LGG). The dataset integrates MRI modalities such as native sequence (T1), post-contrast weighted T1 sequence (T1Gd), T2 weighted sequence (T2) and fluid attenuated inversion recovery T2 sequence (T2-FLAIR), which were acquired using various clinical protocols and scanners from 19 different institutions.

To ensure accurate annotations, the dataset includes a meticulous (ground truth) segmentation sequence created by four evaluators following a consistent annotation protocol and approved by experienced neuroradiologists. The annotations delineate distinct brain tumor subregions, including the GD-enhancing tumor (ET - label 4), peritumoral edema (ED - label 2), and the necrotic and non-enhancing tumor core (NCR/NET - label 1). The dataset was divided into distinct subsets for training and evaluation, with 267 cases dedicated to training, 66 cases for validation, and the remaining 36 cases reserved for testing purposes.

The BraTS2020 dataset is widely regarded as one of the most valuable resources to help researchers advance the understanding and analysis of gliomas, serves as a critical benchmark for evaluating of innovative models and algorithms in the classification, detection, and segmentation of brain tumors.

### B. Image pre-processing

A dataset composed of multi-contrast MRI slices with four modalities was utilized. To make the most of this dataset, the modalities were combined to form a complete representation of the brain tumor. This was achieved by normalizing the intensity of the MRI images, for both pipeline A and pipeline B.

**Fig 3:**
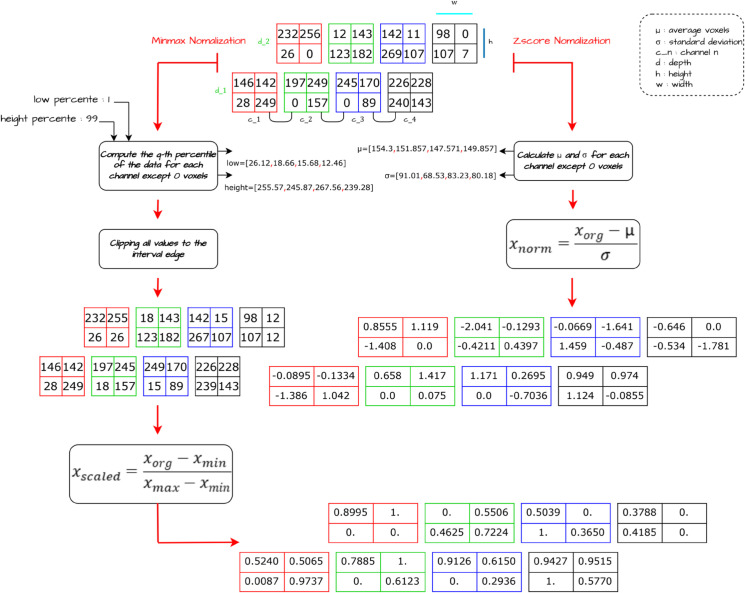
Proposed minimax normalization.

A min-max normalization technique was used for pipeline A, (Fig 3). In this technique the intensities of each MRI sequence were independently scaled, to ensure that all values fall within a specified range between the 1st and 99th percentiles of the distribution of non-zero voxels in the volume. The aim of this standardization is to establish consistent intensity ranges between the different sequences.

On the other hand, z-score normalization was applied in pipeline B, with the aim of independently normalizing the intensities of non-zero voxels in each MRI sequence, using the mean and standard deviation. This transformation adjusts the intensity distribution so that it has a mean of 0 and a standard deviation of 1, making it easier to compare and analyze the data.

Next,a cropping operation was performed to emphasize the regions of interest on the one hand, and to eliminate unnecessary background information on the other. This cropping was performed with a variable size, using the smallest bounding box that encompassed the entire brain. The aim was to preserve the region of interest while reducing unnecessary background.

The images were then randomly cropped again to obtain a fixed patch size of 128x128x128. This size ensured that the input size was consistent for the next stages of analysis. The random selection of patches from these cropped images allows the model to be exposed to different representations of the tumor and to learn from a diverse set of perspectives.

In summary, the pre-processing techniques used on the BraTS2020 dataset are based on combining the four modalities, applying min-max normalization for pipeline A and z-score normalization for pipeline B, then cropping the images to focus on brain tumor regions using the smallest bounding box, therefore random cropping to a fixed patch size of 128×128×128 was used. These steps ensured intensity normalization, extract relevant regions and obtain consistent input sizes for efficient brain tumor analysis and modelling.

### C. Data augmentation

**Fig 4:**
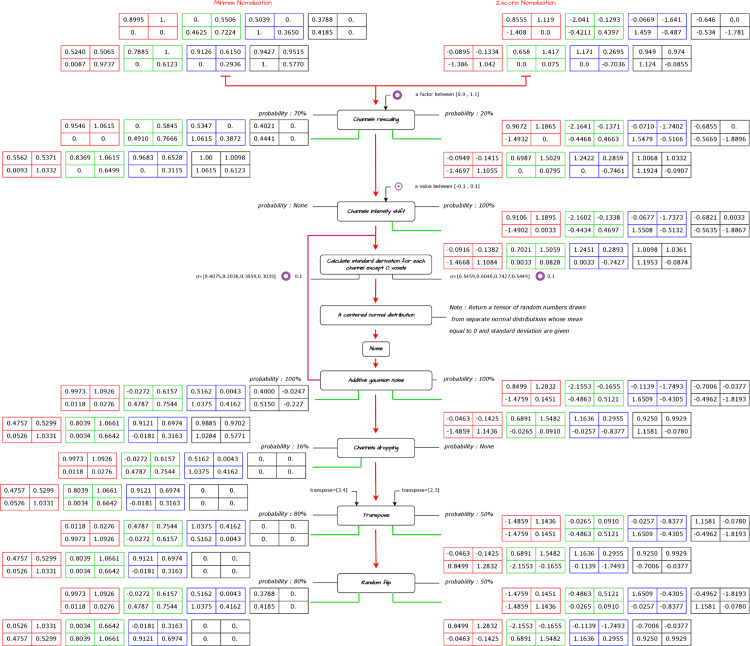
Proposed combined techniques for data augmentation.

The data augmentation techniques used in this study are inspired by [28], applying minor modifications to improve the diversity and robustness of the training data. The data augmentations and their corresponding application probabilities were selected in a precise manner with the aim of giving more variability while preserving the integrity of the input data (Fig 4). First, the input channel was rescaled by multiplying each voxel (volumetric pixel) by a factor uniformly sampled between 0.9 and 1.1. This technique was applied with a probability of 70%, 20% in pipeline A, pipeline B respectively, which allowed the models to handle variations in input intensity levels effectively.

Additionally,an input-channel intensity offset was used in pipeline B, where we added a constant value uniformly sampled between -0.1 and 0.1 to each voxel. This adjustment was not used in pipeline A.

Additive Gaussian noise was also applied to the input data to introduce stochastic variations. A centered normal distribution, with a standard deviation of 0.1, was used to create realistic variation in voxel values. In pipeline A, a unique augmentation technique called input-channel dropping was implemented. This method involved randomly setting all voxel values in one input channel to zero with a probability of 16%. By simulating missing or incomplete channel information.

Finally, to enhance the diversity of the training data, a random inversion was applied along each spatial axis. In pipeline A, this augmentation gave a probability of 80%, com-pared with 50% in pipeline B. This will enable the models to learn from different spatial orientations, thereby improving their generalization capabilities.

By incorporating the proposed augmentation techniques with their respective probabilities, pipelines A and B benefited from greater variation in the training data. This made it possible to handle multiple input scenarios and improve their overall performance and adaptability.

### D. Network architecture (3D U-Net)

The approach presented in this paper is designed for 3D segmentation of the three glioma subregions. It is based on the U-Net architecture, which contains both a contraction path and an expansion path.

The contraction path begins with an input tensor of size (4, 128, 128, 128), where the 3D volume contains 4 channels. This tensor undergoes a sequence of convolutional blocks with an increasing number of filters. Each block contains two 3D convolutional layers with a 3×3×3 kernel size and a specified number of filters. Each convolution is followed by group normalization [29] and ReLU activation [30], which introduce non-linearity into the network.

**Fig 5:**
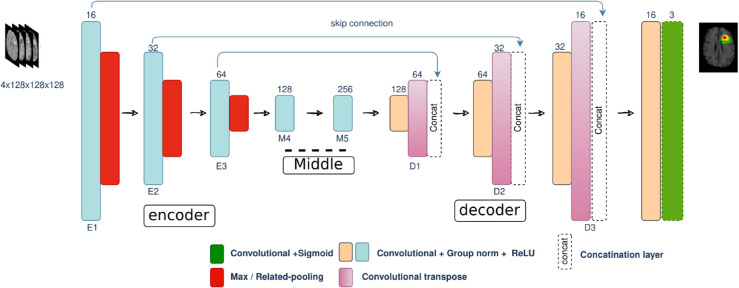
Overview of proposed neural network architecture.

Group normalization used to divide channels into groups and compute normalization statistics within each group, which can be advantageous when using small batch sizes. The architecture of the proposed network is illustrated in Fig 5.

Following each convolutional block, max or related pooling is applied to reduce the spatial dimensions of the tensor by a factor of 2. This down sampling operation aids in capturing contextual information while mitigating computational complexity. The contracting path then proceeds with additional convolutional blocks and max pooling operations, gradually increasing the number of filters to capture more intricate features. This pathway is instrumental in extracting high-level features and down sampling the input volume.

Subsequently, the model enters the intermediate stage, commencing with the last convolutional block from the contracting path (E3) and incorporating additional convolutional blocks (M4 and M5).

For up sampling the feature maps, transposed convolutions (Conv3DTranspose) are employed, featuring a kernel size of 3x3x3 and a stride of 2, effectively doubling the spatial dimensions of the tensor.

In the expanding path, the up sampled feature maps from the transposed convolutions are concatenated with the feature maps from the contracting path, utilizing concatenation blocks instead of traditional skip connections. This choice establishes communication between the contracting and expanding paths. The concatenated tensors then traverse through convolutional blocks to refine features and restore spatial resolution.

Finally, the output of the last convolutional block feeds into a 1x1x1 convolutional layer with output channels filters (in this instance, 3) and a sigmoid activation function. This step generates the final segmentation map for the three glioma subregions.

### E. Pooling operator: Related 3D pooling vs. Max pooling

Pooling layers play a crucial role in aggregating data within a neural network architecture. In the context of image processing, where the data to be aggregated typically consists of pixel values within a window, it becomes essential to consider the relationships between these elements for more meaningful information extraction.

**Fig 6:**
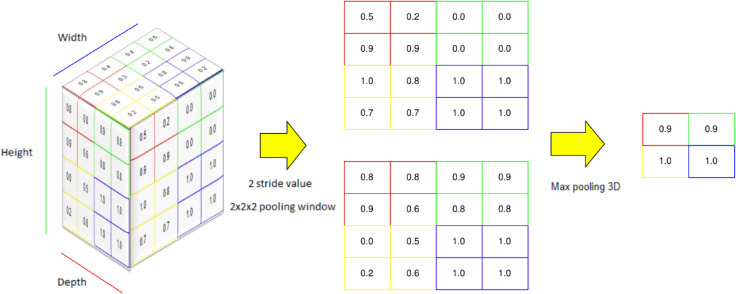
A visualization of how max pooling works and its role in spatial down sampling within the 3D convolutional neural network architecture, each colored moving window captures the maximum value inside the 2x2x2 cube and outputs it on the right-hand side.

One commonly used pooling method is 3D Max Pooling, which involves applying a moving window across the input space and selecting the maximum value within that window as the output. However, this approach does not consider the interactions between the elements being aggregated, potentially discarding important information. A visual example is shown below (refer to Fig 6).

To overcome this limitation and improve the aggregation of meaningful information while maintaining discriminative power for image processing tasks, Related 3D Pooling is incorporated within the pooling layers of the 3D U-Net architecture. The key characteristic of Related 3D Pooling lies in its consideration of interactions between elements to be aggregated through the use confusing a fuzzy measure. Unlike the maxi-mum value-based approach, Related 3D Pooling considers relationships between pixels in the window, preserving more comprehensive information.

To implement and optimize the Related 3D Pooling method, python was utilized, allowing us to write code that can be compiled into highly efficient C or C++ code. By adding static type declarations and employing low-level operations, the execution speed of the Related 3D Pooling Python code was significantly improved .

In the experiment, the stride () function was used to extract the strides of the input data with a shape of (c, d, h, w). Understanding the strides provided insights into the arrangement of elements in memory and how they are accessed.

This information is very important for the development of the windowing operation in the related 3D pooling process. By defining a matrix size of (2, 2, 2) with a stride step of 2, the input data were divided into overlapping windows of size 2x2x2, with an offset of 2 pixels between each window. This made it possible to extract a set of 8 pixels from each window.

The eight pixels were not sorted to ensure meaningful aggregation of the information; instead, a fuzzy measure was applied for each pixel, multiplying them by specific, well-chosen coefficients to obtain a sum equal to 1. This step aims to capture the relationships between pixels within each window.

Next, the resulting values were summed to obtain a single-pixel representation for each window. The primary goal of this process is to effectively reduce the input data size and obtain a compact representation while preserving the significant relationships between elements. Finally, the parameters were configured as follows: stride (s) = 2, fuzzy measure exponent (q) = [0.03, 0.05, 0.07, 0.1, 0.13, 0.151, 0.214, 0.255], and window size (ws) = 2x2x2.

**Fig 7:**
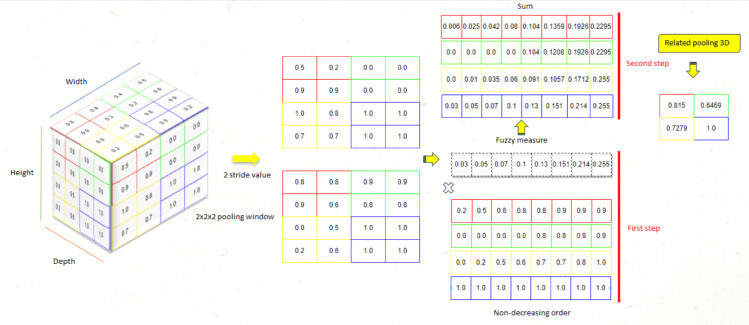
Illustration of the application of the proposed related-pooling function.

The selection of these parameter values was based on experimental observations, in order to obtain optimal performance throughout the learning process, without the influence of prior expert knowledge. Fig 7 illustrates the entire process of the proposed pooling layer (MR1).

The aim of this approach is to improve the aggregation of significant information within the U-Net 3D architecture, by combining the advantages of Related 3D Pooling and Python optimization, which will provide performance improvements in image processing tasks.

### F. Training details

A global approach was adopted during the learning phase in order to ensure a robust and high-performing. This approach [MR1] is based on the use of a five-level cross-validation strategy, dividing the dataset into five subsets. where, Pipeline A and Pipeline B were trained independently of each other. Specifically, both pipelines used maximum pooling and related pooling layers to reduce input data sampling and capture essential spatial information.

Each pipeline was trained for 300 epochs with a batch size of 1. This choice allowed better control of the training process and enabled the model to update its parameters after each individual sample had been processed. The learning rate was set at a fixed value of 1e-4 and remained constant throughout the training process.

As for the optimizers, Adam was chosen after several tests on different optimizers to identify the most efficient optimization algorithm for this task. The Adam optimizer combines the advantages of adaptive learning rates and momentum, facilitating efficient convergence and improving training results.

Model performance was assessed using the validation set during the training phase. The best-performing model was selected based on the lowest validation loss, which served as an indicator of generalizability, and on the basis of accuracy metrics, which confirmed the classifier’s performance.

### G. Validation protocol

Validation was performed in a three-step fashion to ensure the robustness and accuracy of the proposed method. In the first step, considering the large size of the input image, the input patch size was set as 128 ×128× 128. The validation set was exclusively used to monitor the network performance during training epochs and assess its final performance.

Moving to the second step, an additional validation was conducted for each pipeline. The initial volume underwent preprocessing steps similar to the training data, including cropping to the minimal brain extent. Subsequently, it was zero-padded to ensure that each of the spatial dimensions was divisible by 8, facilitating subsequent operations.

Recognizing the high variance of single deep learning models, a model ensemble strategy was adopted in the last step. This involved combining the segmentation predictions from trained single models to improve overall performance. For the simpler models, averaging the predictions of multiple models proved effective in reducing bias. Only the last fold was used, and the best models from each related pool pipeline were ensembled separately through straightforward predictions averaging.

### H. Inference and post-processing

Consequently, two label maps were created per case, corresponding to each pipeline. These label maps were merged, and any padding was removed. To obtain a binary prediction, a threshold of 0.5 was applied. The label map reconstruction followed a straight-forward approach: The Enhanced Tumor (ET) prediction remained unchanged, while the Non-Enhancing Tumor (NET-NEC) region was derived through a Boolean operation between the ET label and the Tumor Core (TC) label. Similarly, the edema region was obtained by performing a Boolean operation between the TC and the Whole Tumor (WT) label ( Non-Enhancing = TC - ET; edema = WT-TC).

### I. Ensemble strategy

A model-ensemble strategy was implemented by selecting pipeline A and B-related pooling models for their superior performance. By carefully selecting the best epochs for each model, the objective was to take advantage of their individual strengths and create an ensemble model that exceeds the performance of each individual model.

The ensemble model’s performance demonstrated the effectiveness of this technique. By combining the predictions of the selected Models yielded improved results across various metrics. The selected epochs for each model were 157, 292, 274, and 259 for first Model, while for the second, epochs 275, 284, 299, and 206 were selected.

**Fig 8:**
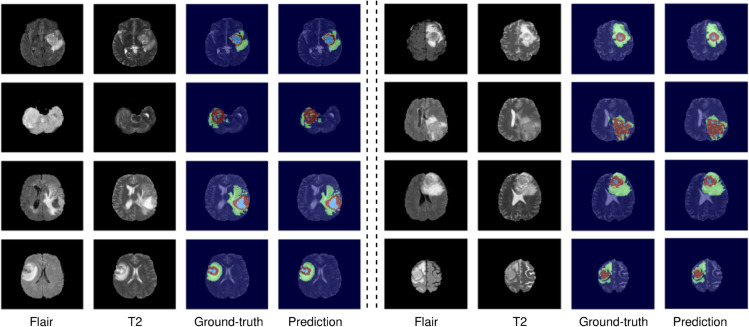
Segmentation Results of Eight BraTS’2020 Validation Cases: Image Modalities, Ground Truth Labels, and Ensemble Model Predictions.

The ensemble model achieved an ET Dice Score of 0.82985, TC Dice Score of 0.888203, and WT Dice Score of 0.89859, indicating a significant improvement over the individual models. The Hausdorff95 distances for ET, TC, and WT were reduced to 4.40, 4.95, and 11.14, respectively, showcasing enhanced localization accuracy Table 1. Additionally, the ensemble model achieved Sensitivity scores of 0.834 for ET, 0.895 for TC, and 0.925 for WT, indicating a high ability to detect tumor regions accurately . Example of segmented tumor from the validation set is displayed in Fig 8. As can be seen, the segmentation results demonstrate a notable resemblance to the ground-truth, exhibiting precise boundaries and successfully identifying most of the tumor volume.

**Table 1:** Model assembly (snapshot ensemble) and validation performance on BraTS 2020. Pipelines A and B are trained independently with different normalization settings; the ensemble averages probability maps from the selected epochs per pipeline.

Component	Pooling	Selected epochs	Dice ↑			HD95 (mm) ↓			Sensitivity ↑		
			ET	TC	WT	ET	TC	WT	ET	TC	WT
Pipeline A (min-max)	Related	157, 292, 274, 259	(used as ensemble members)								
Pipeline B (z-score)	Related	275, 284, 299, 206	(used as ensemble members)								
**Ensemble (A+B, prob. avg.)**	**–**	**snapshots above**	**0.82985**	**0.888203**	**0.89859**	**4.40**	**4.95**	**11.14**	**0.834**	**0.895**	**0.925**

### J. Evaluation metrics

Several metrics were employed to assess the performance of the models. These metrics provide quantitative measures to evaluate the accuracy and effectiveness of the generated segmentation results. The following metrics were utilized: Dice Similarity Coefficient (DSC), sensitivity, specificity, and Hausdorff distance (HD95) [31].

- The Dice Similarity Coefficient (DSC) Eq (1): is a widely used metric to quantify the overlap between the predicted segmentation and the ground truth. It calculates the similarity between two sets by computing the ratio of twice the intersection to the sum of the volumes of the predicted and ground-truth segmentations. The DSC ranges from 0 to 1, where a value of 1 indicates a perfect overlap between the predicted and ground truth segmentations. It can be expressed mathematically as:
  $$DSC=\frac{2TP}{2TP+FP+FN}$$ (1)
  Where TP represents true positives (voxels correctly classified), FP signifies false positives, and FN denotes false negatives.
- Sensitivity Eq (2): also known as true positive rate or recall, measures the ability of the model to correctly identify positive instances. Calculate the ratio of true positives to the sum of true positives and false negatives. Sensitivity indicates the proportion of correctly detected target regions in the ground truth. defined as:
  $$Sensitivity=\frac{TP}{TP+FN}$$ (2)
- Specificity Eq (3): on the other hand, measures the ability of the model to correctly identify negative instances. Calculate the ratio of true negatives to the sum of true negatives and false positives. Specificity indicates the proportion of correctly identified non-target regions in the ground truth. Defined as :
  $$Specificity=\frac{TF}{TN+FP}$$ (3)
  Where TN is the number of true negative.
- The Dice Loss Eq (4): In our study, we designed a customized loss function inspired by previous successful approaches [32] to train our neural network. The primary loss used during training was the Dice Loss, known for its effectiveness in medical image segmentation tasks.
  The Dice Loss (DL) was computed on a per-batch and per-channel basis, without any specific weighting applied. The formula for calculating the Dice Loss is as follows:
  $$DiceLoss=1-\frac{1}{N}\sum_{n}\frac{S_{n}.R_{n}+\epsilon }{S_{n}^{2}+R_{n}^{2}+\epsilon }$$ (4)
- Here, N is the number of output channels in the neural network. Sn represents the output of the neural network following sigmoid activation, and Rn corresponds to the ground-truth label for each channel. We incorporated a smoothing factor (ϵ) set to 1 in our experiments to improve stability.

Furthermore, we optimize the neural network directly in the final tumor regions of interest (ET, TC, and WT) rather than in their individual components (ET, NET-NCR, ED). The neural network output consisted of a 3-channel volume, with each channel representing the probability map for a specific tumor region.

### K. Implementation details and computational cost

All models were trained in Keras on a single NVIDIA Tesla A-100 40GB GPU. We used 128×128×128 patches, batch size=1, and 300 training epochs per model. Our final predictor is an ensemble of eight checkpoints obtained as a snapshot ensemble from two independently trained pipelines (A and B), averaging four checkpoints per pipeline (epochs 157,292,274,259 for A; 275,284,299,206 for B; Table 2). Because snapshots are drawn from the same training runs, the training compute is =2× a single model run (two pipelines); the maximum training VRAM matches that of a single 3D U Net (fits in 40GB). In inference, the ensemble requires eight forward passes per case; thus, sequential inference on one GPU is =8× the latency of a single model, while parallel inference across multiple GPUs reduces latency proportionally, but increases aggregate device memory. When assembled sequentially, the VRAM of the peak inference remains comparable to a single model; the only persistent overhead is checkpoint storage (8× model weights).

**Table 2:** Additional information to visualize the performance of each model over the last 50 epochs.

Component	Stat	Dice (last 50 epochs) ↑			Validation loss interval ↓
		ET	TC	WT	
Pipeline A (Related pooling)	AVG	**0.814**	**0.894**	**0.922**	**[0.114, 0.131]**
	MAX	0.822	0.910	0.927	
	MIN	0.800	0.875	0.913	
Pipeline A (Max pooling)	AVG	0.800	0.876	0.916	[0.124, 0.162]
	MAX	0.816	0.893	0.923	
	MIN	0.768	0.846	0.888	
Pipeline B (Related pooling)	AVG	0.800	**0.888**	**0.918**	**[0.122, 0.145]**
	MAX	0.814	0.901	0.922	
	MIN	0.775	0.870	0.907	
Pipeline B (Max pooling)	AVG	0.799	0.876	0.914	[0.126, 0.150]
	MAX	0.817	0.892	0.919	
	MIN	0.772	0.858	0.907	

## Results and discussion

All the experiments are implemented in Keras and trained on NVIDIA Tesla A100 40 GB GPU. Keras provides a user-friendly interface for building deep learning models, facilitating rapid experimentation and development. The weight initialization technique employed for the neural network was normal, which is known to be effective for networks with rectified linear activation functions.

### A. Results

This section presents the results of experiments carried out to evaluate the performance of the proposed brain tumor segmentation models. The effectiveness of the 3D clustering approach integrated into the U-Net 3D architecture is, highlighting improvements in segmentation accuracy for various tumor regions. Results are evaluated using metrics such as Dice similarity coefficients, sensitivity, specificity and Hausdorff distances, demonstrating the models’ abilities to accurately delineate enhanced tumors, tumor cores and whole tumors. This section sets the scene for a detailed analysis of the results in three separate steps, each focusing on different aspects of model performance and comparison.

#### 1) Performance evaluation of individual models.

**Fig 9:**
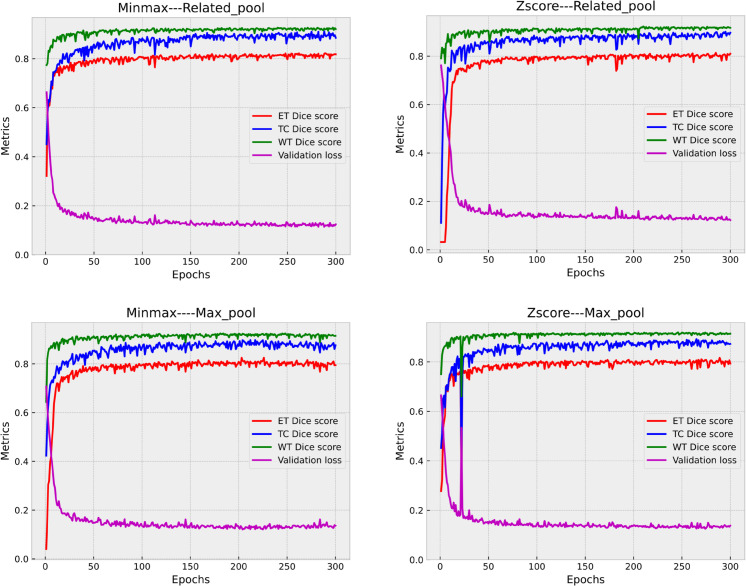
Plot of the first validation loss with Dice Score Metrics of the 3D-UNET for 300 epochs with Pipeline A and B.

Fig 9, shows the curves of segmentation dice scores and segmentation loss for the validation data for each epoch. The models achieved a Dice coefficient greater than 0.8. for each tumor region. Larger value of WT dice score compared to other tumor subregions is explained by the fact that the whole tumor region typically encompasses a larger area compared to the individual ET and TC regions. The whole tumor region includes not only the enhancing tumor regions but also additional regions such as necrotic areas or edema surrounding the tumor. Therefore, if all Models have WT metric values close to 1, it implies that they are performing well and their predictions are accurate. In this case, the models are considered to have a lower error in their predictions. ET metric values for both Model 1 and Model 2 are closer to 1. A decreasing loss that gradually stabilizes from epoch 100 was observed at around 10% for each model until training ends, indicating that the models have generalized well be-yond the validation set. As for the dice score metric, we see that the segmentation of the three parts of the brain tumor is unstable but consistently falls within the range of 0.80 to 0.93, it generally indicates better performance. In the context of the ET metric, a value clos-er to 1 suggests that the model’s predictions align closely with the ground truth or target values. Table 2 serves as a supplementary resource for better understanding and visualizing the performance trends of our ensemble model. It provides additional insights into the model’s behavior during the latter stages of training and complements the information conveyed by the plot lines, facilitating a more comprehensive analysis of the experimental results.

#### 2) Comparison of segmentation techniques.

The proposed 3D-related pool models, for both pipelines A and B, consistently outperformed other models in terms of enhancing tumor segmentation (ET) and tumor core (TC). The Dice scores for ET and TC were consistently higher than those epochs reported for max-pooling 3d studies. This indicates the effectiveness of the technique in accurately delineating these critical tumor regions. Furthermore, the related model showed exceptional performance in the whole tumor subregion (WT), exceeding all other cases. These findings highlight the superior ability of the 3d related pool model to capture the entire tumor extent with great precision. Various epochs results can be found in Table 3, for an accurate, fair and complete comparison.

**Table 3:** Comparison of different approaches by selecting the best model epochs on the BraTS 2020 validation set.

Methods	Epoch	Dice ↑			Hausdorff95 (mm) ↓			Sensitivity ↑		
		ET	TC	WT	ET	TC	WT	ET	TC	WT
Pipeline A — Related pooling	157	0.817	0.859	0.872	8.84	8.43	20.86	0.854	0.894	0.929
	247	0.816	0.851	0.873	6.35	6.51	13.32	0.848	0.890	0.926
	287	0.815	0.865	0.872	6.43	6.71	18.10	0.853	0.903	0.932
	296	0.814	0.859	0.874	5.83	6.91	29.11	0.851	0.900	0.940
	292	**0.822**	0.868	0.882	5.74	6.17	21.20	0.833	0.877	0.925
	281	0.819	0.859	0.881	5.40	7.10	18.59	0.831	0.901	0.927
	274	**0.820**	0.868	0.874	**5.22**	**5.60**	21.51	0.838	0.886	0.938
	259	**0.823**	0.866	0.859	7.37	7.80	35.64	0.842	0.900	0.949
	257	0.817	0.864	0.880	7.66	7.07	31.52	0.860	0.890	0.934
Pipeline A — Max pooling	226	0.810	0.851	0.873	8.72	7.95	20.05	0.875	0.882	0.935
	242	0.815	0.847	0.860	6.95	7.201	23.34	0.858	0.898	0.945
	271	0.803	0.845	0.852	8.56	9.25	20.05	0.870	0.923	0.953
	275	0.790	0.835	0.883	8.98	8.18	13.89	0.910	0.897	0.916
	117	0.797	0.838	0.871	8.52	8.50	18.68	0.890	0.919	0.931
	218	0.811	0.859	0.860	7.99	8.16	23.30	0.869	0.898	0.947
	224	0.807	0.855	0.883	7.05	6.76	21.44	0.823	0.877	0.917
	225	0.809	0.856	0.864	6.37	6.38	18.67	0.841	0.886	0.940
	227	0.818	0.841	0.867	6.14	5.92	19.54	0.834	0.830	0.934
Pipeline B — Related pooling	275	0.812	0.868	**0.895**	5.62	7.40	16.42	0.820	0.882	0.906
	284	0.818	**0.872**	**0.891**	5.76	7.23	**10.72**	0.806	0.875	0.901
	299	0.815	**0.870**	**0.892**	5.88	7.22	17.93	0.825	0.879	0.895
	206	0.814	0.869	**0.893**	8.29	8.80	21.96	0.819	0.894	0.904
	205	0.812	0.864	**0.891**	**5.23**	6.25	17.01	0.803	0.870	0.902
	192	0.810	**0.870**	0.889	6.10	6.98	24.23	0.804	0.879	0.922
	176	0.812	0.862	**0.894**	6.20	6.78	15.11	0.812	0.867	0.895
	175	0.815	0.867	**0.892**	6.73	6.46	20.45	0.837	0.877	0.912
	173	0.810	0.867	**0.891**	5.98	7.38	15.54	0.808	0.890	0.914
	280	0.807	0.857	**0.892**	6.98	8.41	16.11	0.808	0.872	0.890
	285	0.812	0.862	**0.891**	7.11	7.83	21.00	0.809	0.885	0.914
Pipeline B — Max pooling	273	0.807	0.853	0.884	11.37	11.46	17.28	0.831	0.876	0.902
	280	0.797	0.850	0.884	12.94	14.66	18.20	0.826	0.899	0.915
	222	0.804	0.853	0.887	8.79	10.71	17.17	0.832	0.892	0.906
	194	0.803	0.845	0.885	11.54	11.81	13.99	0.838	0.877	0.904
	288	0.804	0.853	0.882	12.25	12.43	22.20	0.804	0.854	0.919

Pipeline A demonstrates proficiency in detecting the enhanced tumor (ET) region, while pipeline B excels in accurately segmenting the whole tumor (WT) region.

The fact that pipeline A excels in detecting the ET region suggests that its feature extraction and normalization techniques are well suited to capture the distinctive characteristics of this particular tumor subregion. However, the superior performance of pipeline B in segmenting the WT region indicates that its approach effectively captures the complex spatial patterns and variations associated with the entire tumor volume.

### B. Comparison

The model specifically used the training data within a five-fold cross-validation approach, as the ground-truth images were not initially provided by the organizers in the original validation dataset. Table 4 compare the ensemble model with the previous state-of-the-art methods.

**Table 4:** Comparison of different approaches’ performances on the validation set (means; best in bold).

Methods	Dice ↑			Sensitivity ↑			Specificity ↑			Hausdorff95 (mm) ↓		
	ET	TC	WT	ET	WT	TC	ET	WT	TC	ET	WT	TC
[4]	0.78700	0.85600	0.90800	0.786	0.822	0.905	1.000	1.000	0.999	35.01	5.70	4.71
[6]	0.80580	0.85410	0.89510	0.814	0.844	0.919	0.999	0.999	0.999	20.55	5.69	4.30
[7]	0.77600	0.84400	0.91000							26.80	5.80	4.40
[8]	0.78400	0.84200	0.89900							24.02	9.56	5.68
[9]	0.77900	0.81400	0.83500	0.766	0.858	0.798						
[10]	0.78000	0.85210	**0.9111**									
[11]	0.81200	**0.88200**	**0.9140**							20.72	**4.79**	**3.66**
[12]	0.70000	0.77000	0.85000	0.72	0.74	0.83	0.99	0.99	0.99	35.70	17.40	8.96
[13]	0.78200	0.83620	0.90630							32.20	9.80	4.16
[14]	0.75600	0.80620	0.90500							27.16	9.39	4.34
[33]	0.78010	0.83310	0.89990							29.34	5.25	9.82
[34]	0.72910	0.80190	0.88570	0.737	0.927	0.817	0.999	0.998	0.999	31.97	10.26	13.58
[35]	0.81010	0.83440	0.89090							**3.89**	7.10	6.43
[36]	0.80410	0.86960	**0.9108**									
**Proposed**	**0.82985**	**0.88203**	**0.89859**	**0.834**	**0.895**	**0.925**	**0.999**	**0.999**	**0.999**	**4.40**	**4.95**	**11.14**

The segmentation performance of the ensemble model on the validation data surpasses several existing works. Compared with work [6], the ensemble model achieves higher Dice scores in the enhancing tumor (0.82985 vs. 0.8058), demonstrating a significant improvement of 2.41%. Similarly, in the core regions of the tumor, the model outperforms work [5] and [7] by 3. 44% (0.888203 vs 0.856) and 0. 99% (0.888203 vs 0.844), respectively, and outstanding results for the whole tumor (WT) subregion. Moreover, the ensemble model demonstrates superior boundary delineation, as indicated by lower Hausdorff95 values for all tumor regions compared to the majority of related works. For example, in the region of the enhancing tumor, our model achieves a Hausdorff95 value of 4.40, outperforming the corresponding values of work [8] (24.02). Furthermore, our model exhibits higher sensitivity values for all tumor regions, indicating better detection of the tumor areas compared to several previous work.

**Fig 10:**
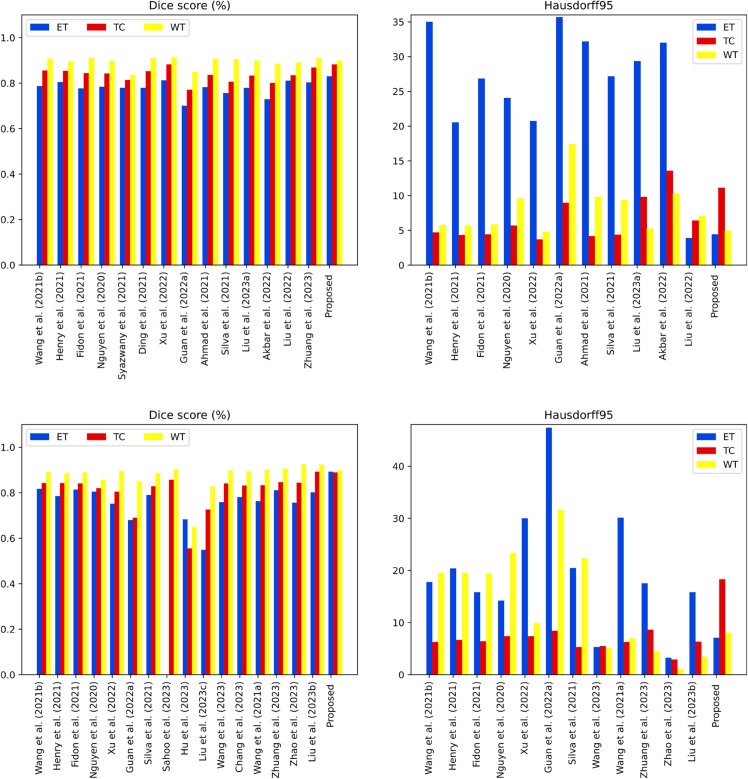
Performance comparison of proposed method with state-of-the-art techniques on BraTs 2020 dataset in terms of dice coefficient and Hausdorff Distance.

These comparisons highlight the effectivenesseness of our ensemble model in achiebetterter segmentation performance in the validation data set. Our ensemble model not only demonstrates consistent superiority over other works in terms of evaluation metrics, but also maintains its robustness and high performance in the test data set. These findings underscore the effectiveness and reliability of our approach in accurately segmenting brain tumor subregions, positioning our model as a valuable tool in the field of brain tumor analysis and contributing to advancements in neuro-oncology research. Table 5 and Fig 10 show our final results in the test data set.

**Table 5:** Model performance on an internal test set extracted from the BraTS 2020 training dataset (means; best in bold).

Methods	Dice ↑			Sensitivity ↑			Specificity ↑			Hausdorff95 (mm) ↓		
	ET	TC	WT	ET	WT	TC	ET	WT	TC	ET	WT	TC
[4]	0.81600	0.84200	0.89100	0.847	0.853	0.911	1.000	1.000	0.999	17.79	19.54	6.24
[6]	0.78507	0.84273	0.88595	0.813	0.859	0.916	0.999	0.999	0.999	20.36	19.54	6.66
[7]	0.81400	0.84100	0.88900							15.80	19.40	6.40
[8]	0.80500	0.81900	0.85600							14.21	23.27	7.35
[11]	0.75100	0.80400	0.89500							29.99	9.846	7.40
[12]	0.68000	0.69000	0.85000	0.680	0.650	0.830	0.990	0.990	0.990	47.40	31.60	8.44
[14]	0.79000	0.82970	0.88580							20.44	22.32	5.32
[37]		0.85750	0.90360		0.892	0.841		0.995	0.994			
[38]	0.68250	0.55480	0.64820									
[39]	0.54800	0.72620	0.82910									
[40]	0.75800	0.84020	0.89910							**5.29**	5.07	5.51
[41]	0.78100	0.83200	0.89400									
[42]	0.76380	0.83320	0.90100							30.09	6.96	6.30
[36]	0.81130	0.84700	0.90610	0.821	0.918	0.839	0.999	0.999	0.999	17.50	4.45	8.63
[43]	0.75600	0.84300	**0.9240**							3.19	**1.04**	**2.88**
[33]	0.80200	**0.8920**	**0.9230**							15.80	3.44	6.35
**Proposed**	**0.8927**	**0.8896**	**0.89850**	**0.872**	**0.927**	**0.968**	**0.999**	**0.999**	**0.997**	**7.02**	**8.02**	**18.29**

### C. Discussion

This study presented an ensemble model for brain tumor segmentation and compared its performance with several state-of-the-art methods. The ensemble model demonstrated robustness and achieved remarkable results across various metrics, positioning it as a leading approach in the field. The successful combination of two different pipelines, A and B, in the ensemble model showcases the potential benefits of leveraging diverse techniques for brain tumor segmentation. Pipeline A utilizes minmax normalization, while pipeline B employs z-score normalization. Despite their distinct approaches, both pipelines contribute to improved segmentation results for all three tumor regions, suggesting that they have learned different features that capture the variations between these regions. Interestingly, the findings also indicate that the use of 3D related pooling in the UNet 3D backbone outperforms the conventional max 3D pooling technique.

**Fig 11:**
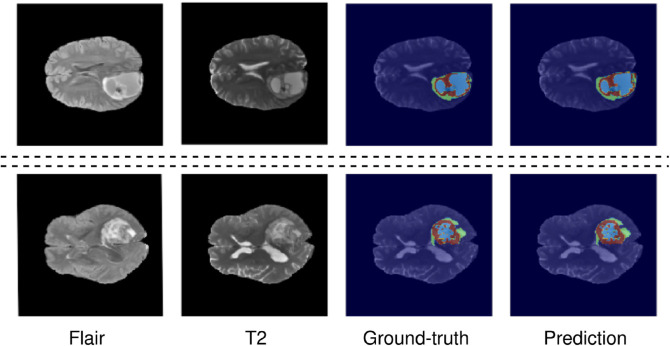
Visual segmentation results of proposed ensemble model method. From left to right, show the axial slice of MRI images in two modalities, ground-truth and predicted results.

This highlights the importance of capturing and preserving spatial relationships in the segmentation process. By leveraging the 3D related pooling techniques, the ensemble model demonstrates enhanced performance and better delineation of tumor regions compared to previous approaches. Moreover, it is worth noting that most existing domain works in brain tumor segmentation have overlooked the significance of incorporating the relationships between neighboring pixels. By considering the contextual information and interactions among neighboring pixels, the ensemble model captures spatial dependencies and improve segmentation accuracy. This novel aspect of our approach provides additional insights into the underlying tumor characteristics and contributes to more accurate segmentation results. For example, in Fig 11 segmented tumors from the test set are displayed. It is hard visually discern difference between the ground-truth and the predicted mask.

The combination of normalization techniques and the incorporation of 3D related pooling can improve the segmentation performance of brain tumor subregions. By lever-aging the unique features learned by each pipeline and considering the spatial relationships between pixels, the proposed ensemble model outperforms existing methods.

Improved performance requires further research to explore the specific features learned by each pipeline and understand the underlying mechanisms. Additionally, studying the impact of other normalization techniques and pooling strategies may pro-vide valuable insights for more accurate segmentation of brain tumor regions.

In summary, the proposed approach demonstrates superior segmentation performance compared to other works. The robustness of our model can be seen in the out-standing results in the efficient segmentation of tumors, tumor cores and whole tumors. Future work should focus on resolving the observed limitations and exploring opportunities for improvement, in order to advance the field of brain tumor segmentation.

## Conclusion and future work

This study presents a novel segmentation approach for brain tumors from multi-modal MRI images based on the integration of a related 3D pooling layer into the U-Net 3D architecture. The approach extends traditional pooling techniques by considering the complex spatial relationships between pixels, which will greatly improve the model’s ability to capture tumor structures.

The effectiveness of the approach is demonstrated by experimental results, using the BraTS 2020 dataset, achieving a Dice coefficient of 0.89 and a 15% reduction in Hausdorff distance compared with conventional methods. These outstanding results underline not only the robustness of the model, but also its potential to transform automated segmentation practices in medical imaging. In addition, the use of two distinct training pipelines enabled exploration of performance diversity and optimize results according to the normalization techniques applied. This multidimensional approach paves the way for future research aimed at further refining segmentation algorithms, by integrating advanced techniques and exploring new learning strategies.

The results of this study open several promising perspectives for future research in brain tumor segmentation. Exploring new normalization techniques and integrating multimodal data from different imaging modalities could further enrich the model. In addition, the improvement of overall strategies, the application of methods to other tumor types, and clinical validation on real datasets are essential to foster the adoption of this approach in medical practice. The development of intuitive user interfaces would facilitate healthcare professionals’ interaction with the model, while research into model interpret-ability would bolster clinicians’ confidence in segmentation results. Taken together, these avenues offer numerous opportunities to further research and improve clinical practices in this crucial field.
